# Unveiling a Hidden Biomarker of Inflammation and Tumor Progression: The 65 kDa Isoform of MMP-9 New Horizons for Therapy

**DOI:** 10.3390/cimb44010008

**Published:** 2021-12-25

**Authors:** Rocco Rossano, Marilena Larocca, Margherita Macellaro, Domenico Bilancia, Paolo Riccio

**Affiliations:** 1 Department of Sciences, University of Basilicata, 85100 Potenza, Italy; laroccamarilena@libero.it (M.L.); margherita.macellaro@gmail.com (M.M.); paoloxriccio@gmail.com (P.R.); 2 Operating Unit, Medical Oncology, Hospital “Azienda Ospedaliera S. Carlo”, 85100 Potenza, Italy; domenicobilancia@gmail.com

**Keywords:** inflammation, matrix metalloproteinases, MMP-9, cancer, metastasis, prognostic markers, urokinase, citrullination, zymography, cancer therapy

## Abstract

Cancer metastasis is a stage of the disease where therapy is mostly ineffective; hence, the need to find reliable markers of its onset. The metalloproteinase-9 (MMP-9, gelatinase B) in its 82 kDa active form, is a good candidate, but here we show that the correspondent little known 65 kDa active MMP-9 isoform, often misrepresented with the other gelatinase MMP-2, is a more suitable marker. Sera from patients with lung and breast cancer were analyzed by bidimensional zymography to detect the activity of MMP-9 and MMP-2. Enzyme identity was confirmed by comparison with MMP-9 standards and by western blotting. The 65 kDa isoform of MMP-9 is a suitable biomarker to monitor tumor progression from tissue neoplasms to metastatic stage, as its activity begins to appear when disease severity increases and becomes very high in metastasis. Moreover, the 65 kDa MMP-9, which derives from the 82 kDa MMP-9, no longer responds to natural MMP-9 inhibitors. As its activity cannot be controlled, its appearance may warn that the pathological process is becoming irreversible. Identification and inhibition of the enzymes converting the inhibitor-sensitive 82 kDa MMP-9 into the corresponding “wild” 65 kDa MMP-9 may allow to develop therapies capable of blocking metastases.

## 1. Introduction

The aim of the present work was to find a reliable laboratory marker of inflammation that may be useful in chronic inflammatory diseases and cancer, particularly in advanced disease states and cancer metastasis.

Indeed, inflammatory mediators are important constituents of the local tumor environment and are present in the systemic circulation [1,2]. In some types of cancer, inflammatory conditions are present before, in others after a malignant change occurs. Thus, in addition to promoting tumor development and survival by promoting angiogenesis and metastasis, inflammatory markers may represent a diagnostic and treatment tool. On these grounds, the control of tumor progression may require a constant monitoring of the inflammatory condition, also in order to evaluate the efficacy of therapy. However, the meta-analysis aimed at the research of gene-markers in cancer, is often associated with contradictory results [3], as nucleic acid measurements are not always well correlated with protein levels [4]. Thus, it is necessary to look for effective markers.

To monitoring the degree of inflammation, we chose as a model tumor progression from tissue neoplasms to the metastatic stage [5], a process associated with a strong inflammatory increase, while, as a biomarker of this model, we chose matrix metalloproteinase-9 (MMP-9 (also called gelatinase B)), an enzyme that is overexpressed in cancer and various inflammatory conditions [6].

In the present study, MMP-9 is not only a marker of inflammation: it has a major role both in opening the way to cancer cells by digesting the extracellular matrix (ECM) all around them and to provide the neo-vasculature necessary to support tumor metastasis [7]. In general, MMPs have a prominent role in preparing the target tissue to invasion by metastatic cancer cells [8,9], and MMP-9 has already been suggested as a biomarker of tumor progression and as a target for anticancer therapy [10,11].

On the Structure and Function of MMP-9

MMP-9 is a member of the MMPs family, 23 zinc-dependent proteolytic enzymes capable of degrading the ECM [12,13], but also other substrates [14].

MMPs are involved in a large variety of physiological processes, such as tissue remodeling, embryogenesis, wound healing, and reproduction [15,16,17]. For this reason, their activity is highly controlled by several mechanisms, also after their expression, as they are secreted as zymogens, have natural inhibitors once activated, and are subjected to various posttranslational modifications (PTM) [17,18], leading to several variants, different in mass and/or electric charge [19]. MMPs are also involved in several inflammatory pathologies including cancer, where in particular MMP-9 favor angiogenesis, cell migration, and metastasis [20].

MMP-9 is a multidomain enzyme, structurally one of the more complex MMPs: from the N-terminal to the C-terminal, we have: (1) a prodomain (keeping the enzyme inactive), (2) the active site, (3) three fibronectin repeats, (4) a zinc2+ binding domain, (5) an O-glycosilated domain, and finally, (6) a hemopexin-like domain, which ensures the binding to inhibitors [13]. In accordance with its complexity, using bidimensional (2-D) zymography, we have highlighted the presence of numerous MMP-9 isoforms, variants, oligomers, and heteromeric complexes [19].

Regarding its activity, MMP-9 is first of all highly regulated at the level of its expression (transcription, mRNA, and translation) in the form of a pre-proenzyme. MMP-9 is then secreted as an inactive enzyme of 92 kDa (proMMP-9) and activated by the removal of the pro-domain by a number of enzymes of its family (MMP-2, MMP-7, MMP-10, and in particular MMP-3, a stromelysin) [21], but also by the system urokinase/plasmin [22], as well as by cathepsin G and K [20]. The resulting activated form, the 82 kDa MMP-9, can be inhibited by its natural inhibitors, called TIMPs (tissue inhibitors of metalloproteinase) [13,23].

Further processing of the 82 kDa isoform consists in the removal of the hemopexin-like C- terminal of MMP-9 giving the 65 kDa isoform of MMP-9 [17,23], which does not respond to the natural inhibitors of MMP-9. The 65 kDa isoform of MMP-9 is poorly understood and it is not known which enzyme determines the cleavage of the C-terminus from the 82 kDa isoform of MMP-9 leading to the formation of 65 kDa.

Due to the similarity of its relative mass (Mr) with that of the activated MMP-2 (64–68 kDa), the 65 kDa isoform of MMP-9 is often confused with MMP-2, so much as to have been considered, mistakenly, an isoform of MMP-2 [24]. MMP-2, gelatinase A, with a Mr of 72 kDa, is the other gelatinase belonging with MMP-9 to the MMPs [13].

However, studies on multiple sclerosis (MS) patients have correctly shown that active MMP-9 can be represented by two different isoenzymes, the 82 and 65 kDa isoforms [25] and that this latter is the predominant active isoform associated with MRI activity in MS [26].

In this paper, we report on the zymographic detection of the serum 65 kDa isoform present in the metastatic phase of breast or lung cancer. This 65 kDa isoform was already observed by Lovett et al. [24] but was attributed to MMP-2. Zymographic detection of MMP-2 and MMP-9 was observed in breast cancer sera as early as 2004 [27], but it is very likely that the increase attributed to MMP-2 at that time was actually that of the 65 kDa MMP-9.

In addition to being a new laboratory biomarker, the 65 kDa MMP-9 isoform opens up new horizons in the study of MMP-9 and may represent a target for therapy, by identifying and blocking the currently unknown enzymatic system that converts the 82 kDa isoform of MMP-9 into its corresponding 65 kDa isoform, as a tumor enters its metastatic stage.

## 2. Materials and Methods

### 2.1. Samples

The sera were obtained from the Hospital ‘San Carlo’ (Potenza, Italy) with the approval (EC code 74/2021) of the Regional Ethical Committee of Basilicata (CEUR of Region Basilicata). The analyses shown in this study are from sera from four patients with lung and breast cancer, as indicated below:

1. Lung cancer at an advanced stage, treated with Opdivo.
2. Lung cancer at an advanced stage, treated with Opdivo.
3. Breast cancer at an early stage treated with paclitaxel adjuvant.
4. Advanced breast cancer with liver metastases treated with docetaxel (taxotere), in combination with pertuzumab (Perjeta) and trastuzumab (Herceptin).

Samples of prostate cancer, gastric cancer, and oropharyngeal cancer were also analyzed, but not shown here.

Before analysis, all samples were centrifuged at 10,000× *g*, 4 °C for 15 min.

### 2.2. Detection of Gelatinases by 2-D Gelatin Zymography (2-DZ)

Gelatinases were detected by bidimensional zymography (2-DZ) [19] by applying samples under non-reducing conditions. Briefly, 100 ng of human recombinant proMMP-9 CHO cells (Calbiochem, Milano, Italy) activated with p-aminophenylmercuric acetate (APMA) or 50 µL of sera was resuspended in the rehydration solution containing 7 M urea, 2 M thiourea, 2% CHAPS (*w*/*v*), and 0.5% (*v*/*v*) IPG (Immobilized pH Gradient) buffer, plus a trace of bromophenol blue, to a final volume of 450 µL. IEF was performed on IPG Dry-Strips of 24 cm with a linear pH gradient of 4–7 (GE-Healthcare, Uppsala, Sweden). IPG Dry-Strips were rehydrated with a sample-containing rehydration solution for 12 h at 20 °C. IEF was run using an IPGphor unit (Amersham Biosciences, Uppsala, Sweden) at 20 °C for a total of 36,000 Vh. After IEF, IPG-strips were equilibrated for 20 min by gentle shaking in equilibration buffer: 6 M urea, 30% (*w*/*v*) glycerol, 2% (*w*/*v*) SDS, 50 mM Tris-HCl (pH 8.8). In the second-dimension proteins were separated, in an 8.5% (*w*/*v*) polyacrylamide gel (20 cm × 24 cm) copolymerized with 0.1% (*w*/*v*) gelatin, on the Ettan DALT II system (Amersham Biosciences, Uppsala, Sweden) at 4 °C first for 30 min at 240 V and then for 6 h at 350 V. Protein Standards Dual Color (Bio-Rad) were used as molecular weight markers. After electrophoresis, gels were washed two times with 2.5% (*v*/*v*) Triton X-100 and then incubated for 14 h at 37 °C in developing buffer (1% *v*/*v*) Triton X-100, 40 mM Tris-HCl buffer (pH 7.0), and 10 mM CaCl_2_. For the development of enzymatic activities, gels were stained with Coomassie Brilliant Blue R-250 (for 30 min at room temperature) and destained in methanol/acetic acid/H2O. The 2-DZ gels were scanned using a ScanMaker 9800 XL-Microtek (Hsinchu, Taiwan). Images were digitally converted from positive to negative images. Determination of apparent Mw and pI was carried out using the ImageMaster 2D Elite V. 2002.01 software (Amersham Biosciences, Uppsala, Sweden).

### 2.3. Western Blot Analysis

A pool of sera from four patients with advanced lung cancer was used for western blot. Pooled sera were desalted and concentrated five-fold to a final volume of 120 µL with 2.0 mL of deionized water in Vivaspin 500, MWCO 30,000 (GE Healthcare, Uppsala, Sweden) and subjected to bidimensional electrophoresis (2-DE). 2-DE analyses were performed using IPG Dry-Strips of 13 cm (linear pH gradient of 4–7) for IEF, whereas in the second dimension, enzymes were separated on 15 cm × 16 cm gel, using the SE 600 Hoefer System. After an electrophoretic run, gels were cut and blotted on a nitrocellulose membrane (for 90 min at 350 mA), using a MINI-trans Blot apparatus (Bio-Rad Laboratories, Segrate, MI, Italy). After transfer, the corresponding nitrocellulose membranes were treated with the following primary antibodies [19]:

- Anti-MMP-9 (Ab-8) mAb (IA5) at a concentration of 2.66 µg/mL (Calbiochem, Milano, Italy). This antibody recognizes both the 92 kDa latent and the 86 kDa active forms.
- Anti-NGAL Ab (5G5) at a concentration of 2.0 µg/mL (EuroClone, Pero, MI, Italy). It recognizes the human NGAL.
- Anti-MMP-9 (4A3) Ab at a concentration of 2.0 µg/mL (Novus Biologicals, Segrate, MI, Italy). It recognizes both the 82 kDa and the 65 kD active forms,
- Anti-MMP-2 Ab (Ab-4) mAb (75-7F7) at a concentration of 1.0 µg/mL (Calbiochem, Milano, Italy). It recognizes the both 72 kDa latent and the 66 kDa active forms.

Thereafter, the membranes were incubated with the HRP-labeled secondary antibody (anti-mouse, Invitrogen). Finally, detection was carried out with ECL reagent (GE Healthcare, Uppsala, Sweden) for detection on X-ray film (GE Healthcare ). Images were acquired using a ScanMaker 9800 XL-Microtek.

### 2.4. Gelatinase Purification

Gelatinase purification was performed by miniature gelatin affinity chromatography [19]. Briefly, 0.5 mL of pooled sera of five lung cancer patients, diluted with 0.5 mL of 50 mM Tris pH 7.5, 0.6 M NaCl (equilibration buffer), were added to mini-spin columns (Bio-Rad Laboratories, Segrate, MI, Italy) loaded with 0.5 mL gelatin-Sepharose 4B beads (GE Healthcare, Uppsala, Sweden) previously equilibrated with equilibration buffer. The mixture was then equilibrated at 4 °C for 60 min (vortexing every 10 min and reversing the column). Successively, the cap was removed and the unbound sample was removed by centrifugation at 3000× *g* (Amicon MC-13 microcentrifuge; Millipore, Bedford, MA, USA), for 2 min. Beads were washed two times with washing buffer 50 mM Tris pH 7.5, 0.1 M NaCl, and gelatinases were eluted with 150 μL of 5% DMSO in 50 mM Tris pH 7.5, 0.1 M NaCl after incubation for 30 min. at room temperature. The eluate was desalted and concentrated to a final volume of 30 μL with 2.0 mL of water in Vivaspin 500 (MWCO 30,000; GE Healthcare, Uppsala, Sweden). A total of 5 µL of purified gelatinase was applied for 2-DZ analysis, whereas 25 µL of sample was subjected to 2-DE analysis for western blot identification of 65 kDa MMP-9 active form.

## 3. Results

### 3.1. Identification of MMP-9 on Zymograms

**Figure 1:**
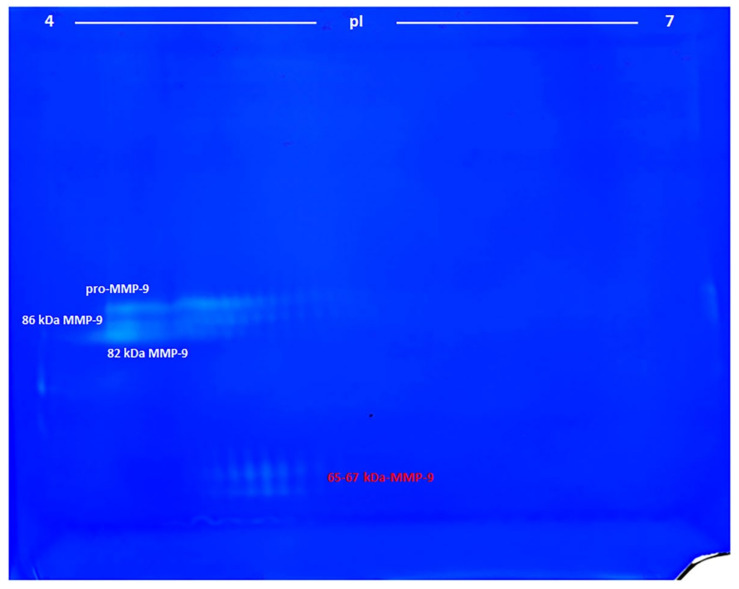
Bidimensional zymography (2-DZ) of MMP-9 standard. A total of 100 ng of human recombinant proMMP-9 CHO cells (Calbiochem) activated with p-aminophenylmercuric acetate (APMA) was resuspended in the rehydration solution and subjected to isoelectrofocusing (IEF) on IPG Dry-Strips of 24 cm in a linear pH gradient of 4–7 (first dimension) and then applied for the second dimension in a 8.5% (*w*/*v*) polyacrylamide gel (20 cm × 24 cm) copolymerized with 0.1% (*w*/*v*) gelatin.

Since the marker proposed in this paper is attributed to MMP-9 and not to MMP-2, and the analysis is performed by zymography, we had first to verify if it is present in the commercially available MMP-9 standard. To this end, the human recombinant proMMP-9 CHO cells from Calbiochem was analyzed by 2-DZ, after activation with p-aminophenylmercuric acetate (APMA) (Figure 1). Zymograms revealed the presence of several spots (clear, unstained spaces), indicating the presence of gelatinolytic activity, as detailed below. From top to bottom, four groups of spots were detected:

1) The pro-MMP-9—a broad spot around Mw 90–92 kDa and pI 4.2–5.0;
2), 3) The activated MMP-9—two groups of spots of about 86 and 82 kDa and pI range of 4.2–5.0 and 4.2–4.6, respectively;
4) The 65–67 kDa MMP-9 active form—a group of spots with pI range 4.6–4.9, corresponding to the marker suggested in this work.

### 3.2. Lung Cancer

**Figure 2:**
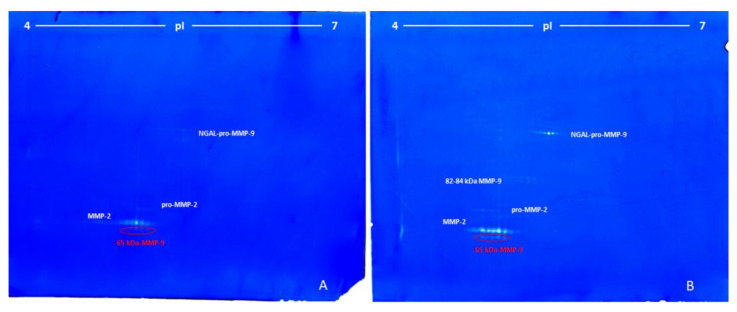
Bidimensional zymography (2-DZ) of sera from patients with lung cancer. Gels (**A**,**B**): 2-DZ of serum from two patients with advanced lung cancer treated with Opdivo. For both, aliquots of 50 µL of sera were resuspended in the rehydration solution and subjected to isoelectrofocusing (IEF) on IPG Dry-Strips of 24 cm in a linear pH gradient of 4–7 (first dimension) and then applied for the second dimension in a 8.5% (*w*/*v*) polyacrylamide gel (20 cm × 24 cm) copolymerized with 0.1% (*w*/*v*) gelatin.

The gelatinolytic activities, present in the serum from two patients with advanced lung cancer, were detected by non-reducing 2D gelatin zymography (Figure 2A,B), as in the analysis of standard MMP-9, shown in Figure 1.

The zymographic analysis of the serum from the first patients showed four groups of spots with apparent Mr between 65 and 120 kDa and pI 5.11–5.72 (Figure 2A), as follows:

(1) The first group was made of some unresolved spots around 120 kDa and pI between 5.50 and 5.72 (ascribed to the complex NGAL-pro-MMP-9).
(2) The second group (three spots) was located at around 72 kDa and pI 5.25–5.40 (pro-MMP-2).
(3) The third group, characterized by the highest proteolytic activity, consisted of seven spots of 68 kDa at pI between 5.11–5.45 (MMP-2).
(4) The fourth and last group was made of three spots at 65 kDa and pI between 5.20 and 5.35 (65-kDa MMP-9 active form).

Figure 2B shows the 2-DZ zymographic pattern related to the serum of the second patient with advanced lung cancer. The zymographic patterns were similar to those observed previously (Figure 2A), but with some differences. First of all, the 82 kDa MMP-9 was also present with a double row of spots at 82 and 84 kDa and pI ranging from 5.40 to 5.70. Overall, the zymographic profile was characterized by a higher intensity of the digestion spots in the same three zones of the gel observed in Figure 2A.

### 3.3. Breast Cancer

**Figure 3:**
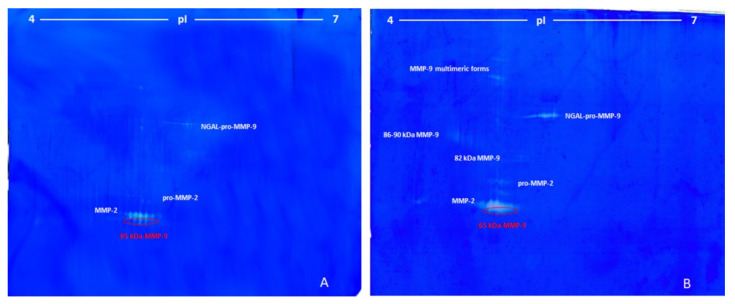
Bidimensional zymography (2-DZ) of sera from patients with breast cancer. Gel (**A**): 2-DZ of serum from patient with breast cancer at an early stage treated with paclitaxel adjuvant. Gel (**B**): 2-DZ of serum from patient with advanced breast cancer with liver metastases treated with docetaxel (taxotere), in combination with pertuzumab (Perjeta) and trastuzumab (Herceptin). For both, aliquots of 50 µL of sera were resuspended in the rehydration solution and subjected to isoelectrofocusing (IEF) on IPG Dry-Strips of 24 cm in a linear pH gradient of 4–7 (first dimension) and then applied for the second dimension in a 8.5% (*w*/*v*) polyacrylamide gel (20 cm × 24 cm) copolymerized with 0.1% (*w*/*v*) gelatin.

Figure 3 shows a representative 2-DZ of sera from patients with breast cancer at an early stage (3A) and with advanced breast cancer with liver metastases (3B).

Zymograms of the sera from breast cancer at an early stage (Figure 3A) were very similar to those observed for the serum of the patients with lung cancer shown in Figure 2A, except for the higher activity of MMP-2 and 65 kDa MMP-9. Conversely, the zymographic pattern of advanced breast cancer with liver metastases (Figure 3B) differed considerably from the breast cancer at an early stage (Figure 3A), as in the advanced state there were three additional groups of spots. The first two group of spots in the region at high molecular weight were ascribed to the multimeric forms of MMP-9, with apparent Mr of 180 and 200 kDa, while a third streaked spot with Mr 86-90 kDa and pI 4.6–4.9 was ascribed to pro-MMP-9 and active MMP-9. Furthermore, in correspondence of the NGAL-pro-MMP-9 and in the region between 65 and 68 kDa, a higher gelatinolytic activity was observed. In the latter case (Figure 3B), the MMP-2 and the 65 kDa MMP-9 were detected as a unique unresolved digestion zone.

### 3.4. Identification of MMP-9 and MMP-2 by Western Blot Analysis

**Figure 4:**
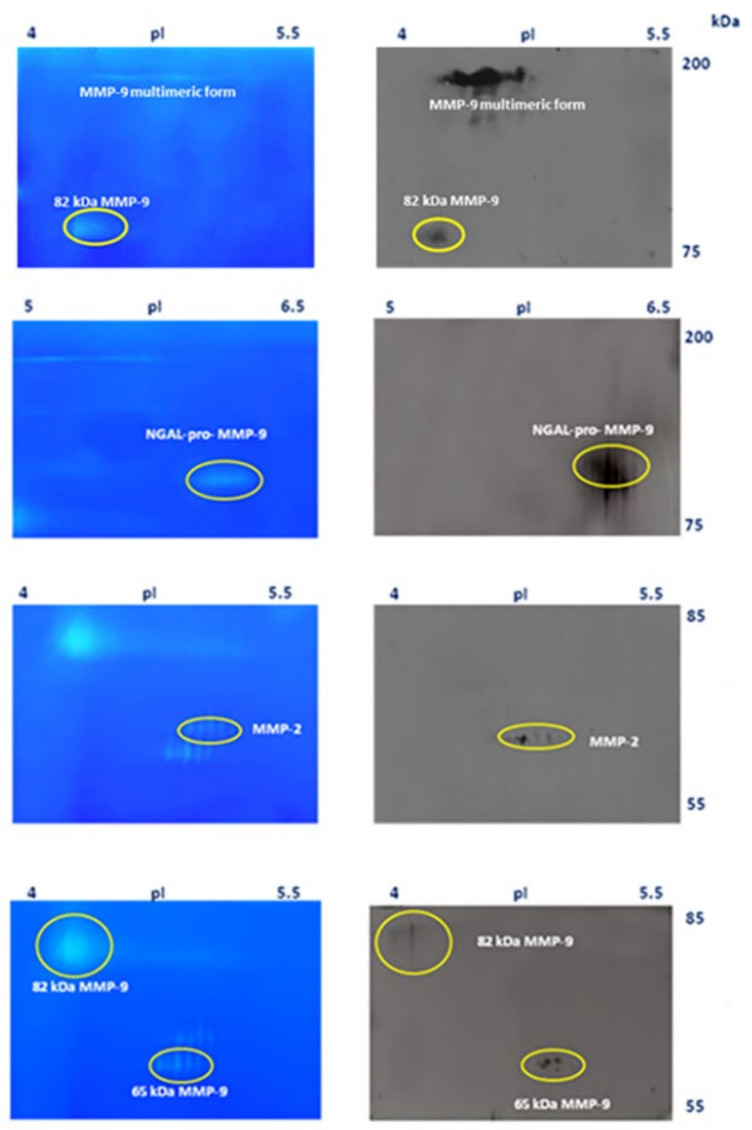
Identification of gelatinases by western blot analysis. A pool of sera from four patients with advanced lung cancer was used. For 2-DZ, 30 µL of pooled sera was applied (**left panels**), whereas for western blot analysis, pooled sera were desalted and concentrated five-fold (Vivaspin 500, MWCO 30,000; GE Healthcare, Uppsala, Sweden) and 30 µL was subjected to each 2-DE (**right panels**). Bidimensional analysis were carried out using IPG Dry-Strips of 13 cm (linear pH gradient of 4–7) for IEF, and 15 cm × 16 cm polyacrylamide gel for the second dimension. 2-DE gels were cut and the corresponding nitrocellulose membranes were treated with anti MMP-9 (Ab-8) mAb (IA5) at a concentration of 2.66 µg/mL, anti-NGAL Ab (5G5) at a concentration of 2.0 µg/mL, anti-MMP-2 Ab (Ab-4) at a concentration of 1.0 µg/mL, and anti-MMP-9 (4A3) Ab at a concentration of 2.0 µg/mL, recognizing the 65 kD and the 82 kD activated forms. Gels shown the region with isoelectric point between pH 4–5.5 and 5–6.5, respectively and molecular mass between 200–75 and 85–55 kDa, respectively.

MMPs were identified by western blot analysis of a pool of sera from four patients with advanced lung cancer, as described above in Section 2 (Figure 4).

In the left panels of Figure 4 are shown the zymographic patterns corresponding to the 2-DE gels blotted on nitrocellulose membrane (right panels of the same figure). From top to bottom, the first panel corresponds to the treatment with MMP-9 (Ab-8) mAb (IA5) antibody, the second corresponds to the membrane treated with anti-NGAL Ab (5G5), the third corresponds to the treatment with anti-MMP-2 Ab (Ab-4), and finally, the bottom panel shows the blot after treatment with anti-MMP-9 (4A3) Ab, recognizing both the 65 kD and the 82 kDa activated forms.

**Figure 5:**
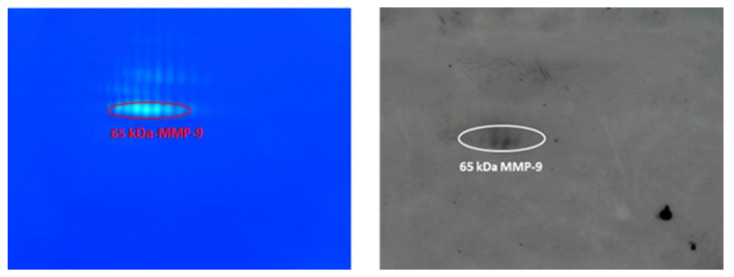
Identification of the 65 kDa MMP-9 form by western blot analysis of a purified and concentrated serum pool from lung cancer patients. Gelatinases in pooled sera were purified by miniature gelatin affinity chromatography [19,25]. For 2-DZ, 5 µl of purified gelatinases was applied (**left panels**), whereas 25 µL of sample was subjected to 2-DE analysis. For western blot analysis, 2-DE gels were cut and the corresponding nitrocellulose membranes were treated with anti-MMP-9 (4A3) Ab (**right panel**).

To further validate the western blotting results shown in Figure 4 and make them more visible, the pool of sera from patients with advanced lung cancer was passed through a small Sepharose gelatin column, to concentrate the gelatinases, as described in [16,25]. MMPs were eluted with DMSO as indicated in the Materials and Methods section and applied to 2-DZ and also to 2-DE for western blotting. Both are shown in Figure 5.

## 4. Discussion

In this paper, we want to highlight a particular isoform of MMP-9, as a biomarker of progression from neoplasm to metastasis. This isoform of MMP-9 is the 65 kDa MMP-9, one of the two main forms of active MMP-9.

As described in the introduction, the two active forms–the 82 and the 65 kDa MMP-9–derive from the MMP-9 pro-enzyme by cutting the N-terminal part (thus giving the 82 kDa MMP-9) and, subsequently, the C-terminal part (with the consequent formation of the 65 kDa isoform) [17,21,23]. Since the C-terminal part is the one that allows the binding of natural MMP-9 inhibitors, the activity of 65 kDa is uncontrollable, unlike the 82 kDa, which is involved in various physiological processes and whose activity must, therefore, be carefully controlled. Hence, the involvement of the 65 kDa in physiological processes is highly unwanted: indeed, inhibition of the 65 kDa at the catalytic site (apparently the only site available to block the enzyme) would obviously also block the 82 kDa isoform and, thus, also the physiological activities.

Regarding the importance of MMP-9 as a biomarker, the 82 kDa isoform has already been considered in several forms of cancer [4,6,9,28,29], while the 65 kDa isoform has escaped the attention of researchers for the reasons given in this work. The only pathology in which 65 kDa MMP-9 has been suggested as a biomarker is, to our knowledge, multiple sclerosis (MS) [25,26].

In one of our previous works, we showed that both MMP-9 and MMP-2 increase in MS [28]. Nowadays, we are aware that the MMP-2 observed at that time was probably the 65 kDa MMP-9. Indeed, at that time [30], its identification as 65 kDa MMP-9 could not be achieved using zymography alone (as also stated in [31]).

However, it should be noted that MMP-9 is not specific to a particular disease: in fact, MMP-9 is more a marker of inflammation, being associated with the activation of the pro-inflammatory transcription factor NF-kB [32]. Indeed, the increase of MMP-9 expression and activity correspond to an increase of the inflammatory state in pathology, so much so that it has even been recently proposed, together with other NF-kB-dependent biomarkers, as a signature associated with mortality in COVID-19 patients [33].

Ultimately, the presence of 65 kDa MMP-9 represents an uncontrollable phase of the disease in question and in general of the inflammatory process associated with it. As long as this isoform of MMP-9 is present, vigilance is needed, as its presence may represent a valid signal of irreversibility for several cancer types.

Here, we want to stress that, although we present in this work only data involving both breast and lung cancer, as the two cancers may be related [34], we found the presence of the 65 kDa MMP-9 also in prostate cancer, gastric cancer, and oropharyngeal cancer samples. The only possibility of blocking the 65 kDa MMP-9 is to inhibit its synthesis from the 82 kDa MMP-9. However, what needs to be inhibited and how? We need to know how the 65 kDa is formed before we can answer this question.

### 4.1. On the Origin of the 65 kDa MMP-9 and the Inhibition of Its Formation

Since the activity of 65 kDa cannot be directly blocked, it is of fundamental importance to clarify which enzyme directly cuts the C-terminus of the 82 kDa MMP-9, giving rise to the 65 kDa MMP-9. This enzyme is not yet known and only hypotheses can be made, and these have yet to be tested.

As far as we know, there are three suitable candidate enzymes for the formation of the 65 kDa MMP-9 from the corresponding 82 kDa isoform: (1) the urokinase system, (2) the peptidyl arginine deiminases (PAD), responsible of the citrullination of MMP-9 [17], and (3) cathepsin K. All are associated with the tumor and metastatic process and (1) and (3) are able to cleave the 82 kDa MMP-9 to give the corresponding isoform of 65 kDa.

### 4.2. The Urokinase System

The first candidate could be the Urokinase-type Plasminogen Activator (uPA), a serin protease involved in the activation of plasminogen to plasmin and in the degradation of the extracellular matrix, thus favoring tumor cell migration from the primary to a secondary site. uPA is a good candidate because it is able to act on MMP-9 as a substrate and because it plays a major role in tumor progression and metastasis [35]. In fact, it increases in the metastatic stage and may represent itself a potential prognostic biomarker to predict poor survival [36].

Thus, the inhibition of the urokinase system could be the key to block both the formation of the 65 kDa MMP-9 and the metastatic process. In this respect, it has been shown that a mixture of lysine, proline, ascorbic acid, and green tea extract might be useful to inhibit uPA [37,38].

In our experience, green tea extracts inhibit both MMP-9 and MMP-2 (hence, also the 65 kDa MMP-9) [39] and a mixture of polyphenols with different structure is effective at very low concentration [40].

Furthermore, both anti-inflammatory diets and dietary supplements could decrease production of MMP-9 and, hence, of the 65 kDa MMP-9, as shown in multiple sclerosis [41].

### 4.3. Peptidyl Arginase Deiminases (PAD) and MMP-9 Citrullination

There are five PAD enzymes. In the presence of Ca2+, PAD enzymes convert the arginine present in some proteins into citrulline [17]. Among these, PAD4 is overexpressed in many tumor tissues and repress p53 (a well-known tumor suppressor) by histone citrullination. It has recently been reported that citrullination or hypercitrullination by PAD2 of proMMP-9 makes proMMP-9 more sensitive to the proteolytic action of MMP-3 [17]. MMP-3 is the enzyme that normally converts proMMP-9 to its 82 kDa active form by cutting the N-terminus. However, if the proMMP-9 is hypercitrullinated, MMP-3 also cuts the C-terminus, giving rise to the smaller MMP-9, with uncontrollable activity. However, it must be considered that the observed product was 57 kDa [17] and not 65 kDa.

The role of citrullination in pathology has been well revisited recently [42]. In addition to cancer, citrullination is associated with several pathological conditions, such as rheumatoid arthritis, systemic lupus erythematosus, and multiple sclerosis [42]. In conclusion, in the case of MMP-9 citrullination, the enzymes to be inhibited would be PADs, not MMP-3.

### 4.4. Cathepsin K

The third enzyme we propose, perhaps apparently a less suitable candidate, is Cathepsin K, a lysosomal cysteine protease. Cathepsin K is able to cleave and activate pro-MMP-9 [20] and is involved in inflammation and in some types of cancer. In particular, Cathepsin K expression is higher in breast cancer and tumor invasiveness in bones, as it is involved also in osteoporosis [43]. However, we found no indication that cathepsin K acts on the 82-kDa isoform by cutting the C-terminal and releasing the corresponding 65-kDa isoform, and therefore, it remains an alternative candidate.

## 5. Conclusions

In this study, we introduce a novel marker of inflammation, which may be useful to monitor the tumor process and catch the onset of metastasis early. This marker is usually overlooked because zymography and ELISA analysis do not allow to distinguish it from the 64–68 kDa MMP-2. The two enzymes can be distinguished from each other by means of ConA Sepharose chromatography, which recognizes their different glycosylation [19,25].

In addition to being a good marker of the metastatic process, the discovery of the 65 kDa isoform of MMP-9 opens up new therapeutic perspectives targeting the enzymes that produce it. In fact, the formation of the 65 kDa isoform is a definitive choice: once formed, its activity can no longer be specifically controlled.

## Data Availability

In the Results section all the data produced are shown.
